# Modeling the Evolution of Collective Synchrony

**DOI:** 10.1111/nyas.70226

**Published:** 2026-03-03

**Authors:** Guy Amichay, Ruoming Gong, Daniel M. Abrams

**Affiliations:** ^1^ Department of Engineering Sciences and Applied Mathematics Northwestern University Evanston USA; ^2^ Northwestern Institute for Complex Systems Northwestern University Evanston Illinois USA; ^3^ National Institute for Theory and Mathematics in Biology Northwestern University Chicago Illinois USA; ^4^ Department of Physics and Astronomy Northwestern University Evanston Illinois USA

**Keywords:** collective synchrony, coupled oscillators, evolutionary game theory, Kuramoto, lek, social cheating

## Abstract

Group synchrony in the animal kingdom is usually associated with mating. Being in sync is likely advantageous, as it may help in luring the opposite sex. Yet there are also disadvantages—such as the homogenization of the group—which make it harder for individuals to stand‐out. Here we address this trade‐off, bringing together the Kuramoto model with concepts from evolutionary game theory. We focus on the existence of self‐interested cheaters, which have been extensively studied in a variety of species. In our scenario, cheating individuals take part in the synchronous group display but position themselves (in terms of phase) slightly ahead of or behind the pack. This allows them to enjoy both the group benefit of advertisement and the individual benefit of being unique. But a group can only tolerate a limited number of such individuals while still achieving synchrony. We therefore incorporate a form of policing into our model: If an individual strays too far form the group's synchronous phase, they reveal themselves as dishonest and are punished. Our model offers testable predictions regarding natural population compositions, and will hopefully spur further investigation into not only how, but also why, natural systems synchronize.

## Graphical abstract

We model synchronized mating displays as a Kuramoto‐style evolutionary game with honest cooperators and phase‐shifted cheaters. Cheaters exploit group advertisement while remaining distinctive, but excessive deviation triggers policing. Our analysis shows stable coexistence with an upper bound on the fraction cheating, and predicts that only symmetric enforcement of early and late signalers sustains long‐term synchrony in animal groups.

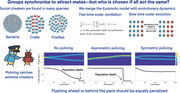

## 1 Introduction

Synchrony plays a crucial role in the behavior of many animals, often manifesting as coordinated acoustic or visual signals [1]. In many cases, synchrony is thought to emerge due to the evolutionary advantages it provides, as opposed to simply being epiphenomenal. A well‐known example is synchronization in mating displays, where coordinated flashing or calling can amplify signals, making them more effective for attracting mates over longer distances (the “beacon hypothesis” [2]) (though of course collective courtship lekking displays are not necessarily synchronous [3, 4]).

For instance, certain species of male fireflies exhibit synchronized flashing, which is thought to help attract females from afar [2]. Yet understanding this remains a challenge: If all individuals flash simultaneously, how can any one of them stand out to a potential mate? Similar behavior is observed in fiddler crabs, where males wave their claws in synchrony, especially in the presence of females. Strikingly, some species even exhibit this behavior in the absence of females [5], suggesting that the function of synchrony may go beyond reproductive signaling. These examples highlight the need to better understand the evolutionary origins and stability of collective synchronization.

Note that here, when we describe signals as synchronous, we refer to temporal matching of the signal phases,^1^ which also requires frequency locking (i.e., matching of tempos) as a precondition. Frequency locking without phase matching can be seen as a looser form of synchrony that we do not consider here.

Empirically, getting traction on these questions poses multiple problems. Precise measurement of these behaviors in natural ecological contexts is challenging [6, 7]; automatically detecting where and when mating events occur adds another layer of complication. Without such data, though, it is difficult to gain insight into which males are sexually selected for.

We thus focus our efforts here on theory, with the hope that predictions will later be tested against field data. Mathematically, the Kuramoto model has long served as a foundational framework for studying synchronization in large populations [8, 9]. When one accounts for the individual cost of synchronization, it becomes natural to frame the problem using the language of mean‐field games [10, 11, 12, 13]. In this approach, each individual seeks to minimize a cost function, and the system settles into a Nash equilibrium that balances synchronization benefits and individual costs. However, standard mean‐field game approaches usually fall short with regard to long‐term trade‐offs, as they only provide the Nash equilibrium for the current population, and do not say anything about how the population will evolve. As such, the resulting equilibrium does not necessarily reveal which synchronization strategies are preferred or stable over evolutionary time.

Evolutionary game theory provides another lens for studying this problem [14, 15], allowing us to examine which synchronization strategies will be favored and adopted at equilibrium. This approach emphasizes the role of payoff structures in shaping strategy. Yet, in many cases, the choice of the payoff matrix lacks a principled justification, limiting the explanatory power of the framework.

Here we combine the Kuramoto model with an evolutionary game‐theoretic framework to investigate the emergence and stability of synchrony in animal groups. Inspired by dishonest behavior (“cheating”) found in quorum sensing bacteria [16], fiddler crabs [17], or even firefly “femmes fatales” [18], we imagine a scenario where cheaters exist within synchronous groups. Cheating in this context, however, would be different from those previous observations. It would mean taking part in the display, but positioning oneself as an outlier with respect to phase: that is, signaling with some phase advance or delay relative to the group. This would allow the cheaters to, on the one hand, appear as cooperative (they are taking part in generating the collective signal), but to also stand out—potentially giving them an advantage relative to the rest. We ask if, and how, they could play a part in the synchronous dynamics.

Finally, we also introduce the concept of policing (found, e.g., in bacteria [19] and honeybees [20]). Without policing, there may be no incentive to cooperate, so this is a key ingredient to stabilizing synchronous dynamics on an evolutionary time scale. As cheaters can hamper collective benefits, cooperating individuals detecting such activity may enforce the rules—though we do not specify here how this may be achieved (it could be manifested differently in different species). As such, when cheaters become too obvious (e.g., they call antiphase to the rest of the group), they become susceptible to detection and punishment.

Being in a group typically is protective due to the confusion effect [21, 22, 23, 24], though it is not yet well known how such dynamics play out in synchronous collectives. Predation [25], in our case, could have an identical effect to policing. Being too different from the collective will make an individual stand out and be more susceptible to negative consequences that reduce the chances of reproduction. In some cases, though, predation is known to play little to no role—for example, some firefly species are known to be poisonous and thus less predated [26].

## 2 Methods

Our models incorporate two key time scales: a fast (within‐generation) time scale representing the oscillatory behavior of an individual during their lifespan, and a slow time scale representing multigeneration evolutionary processes.

For the fast time scale, we assume that each individual's behavior follows the Sakaguchi–Kuramoto model [27] (a modified Kuramoto model [9, 28]):

(1) $$\frac{d}{dt}\theta_{i}=\omega_{i}+\frac{K}{N}\sum_{j=1}^{N}\sin\left(\theta_{j}-\theta_{i}+\alpha_{i}\right),$$

where $\theta_{i}$ is the phase of individual i, $\omega_{i}$ is its intrinsic frequency (which we will take to be universal), K is the coupling strength, and $\alpha_{i}$ represents the individual's phase lag strategy. When $\alpha_{i}=0$ for all individuals, we recover the classical Kuramoto model, which exhibits a phase transition from incoherence to synchrony as coupling strength K increases. For near‐identical oscillators, as we consider here, only minimal coupling is necessary to produce very highly synchronized states. See [28] for more about the Kuramoto model.

For simplicity, we assume that the strategy of each individual is fully characterized by its phase lag parameter $\alpha_{i}$. A phase lag of zero would mean that the individual dynamics are geared toward in‐phase relationships with neighbors. A nonzero phase lag pushes the individual to be ahead or behind its neighbors in terms of phase (though a system with identical individuals with nonzero phase lag, for example, could still yield synchrony). Phase lags were considered in prior works [29], but were treated as a proxy for delays; here, we reinterpret the meaning, and suggest that this acts as a built‐in strategy to aim to be different.

**FIGURE 1:**
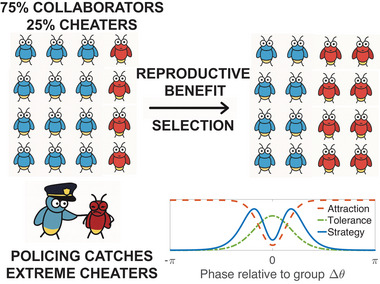
Modeling approach. Upper part shows a schematic that depicts our modeling process: In each generation the group size stays fixed, but the relative amount of cheaters may change due to selection. Note that we use fireflies in this example, but the model is relevant also to other similar systems such as fiddler crabs, frogs, crickets, etc. The lower panel shows how individuals strategically act. The red dashed line is the “attraction function,” the green dot‐dashed line is the policing “tolerance function” (which may also relate to predation) and the blue solid line is the overall “strategy function.” In the mating context, individuals that deviate from the group have a higher chance of being selected, so the attraction function reaches its minimum at Δθ=0. By contrast, policing (the inverse of tolerance) targets individuals who deviate too far, leading the tolerance function to be maximized at Δθ=0. The strategy function, proportional to the product of the attraction and tolerance functions, illustrates the strategies that yield the highest individual benefit.

On the slow time scale, we model natural selection as optimizing a net payoff to each individual. That payoff is dependent on both group‐level and individual‐level properties, and is expressed as the difference between a benefit and a cost. The benefit $b_{i}$ for individual i is defined as the product of two terms: $g_{i}$, the expected number of females attracted to the group (determined by group‐level synchrony); and $f_{i}$, an individual “strategy function,” which quantifies the value of an individual's flash timing strategy. We assume $g_{i}=g_{i}\left(R\right)$ is a positive increasing function of the order parameter R defined as

(2) $$Re^{i\phi }=\frac{1}{N}\sum_{j=1}^{N}e^{i\theta_{j}},$$

where R∈[0,1] quantifies the level of synchrony, and ϕ is the average phase. The strategy function $f_{i}=f_{i}\left(\theta_{i}-\phi \right)$ is a function of phase difference $\theta_{i}-\phi$,^2^ and is taken to be a product of an “attraction” and a policing “tolerance” function, as illustrated in Figure 1. It captures both how individual timing relative to the group influences mate attraction, and how standing out from the group might be tolerated by policing:

(3) $$\begin{array}{c} b_{i}=g\left(R\right)f_{i}\left(\theta_{i}-\phi \right)=g\left(R\right)\left[\mathrm{att}\left(\theta_{i}-\phi \right)\mathrm{tol}\left(\theta_{i}-\phi \right)\right]. \end{array}$$

Policing enables the group to act against extreme (or obvious) cheaters, reducing their payoffs as a result.

On the cost side, letting

(4) $$u_{i}=\frac{K}{N}\sum_{j=1}^{n}\sin\left(\theta_{j}-\theta_{i}+\alpha_{i}\right)$$

denote the active control or effort exerted by individual i, we define the individual cost $c_{i}$ over a time window [0,T] as

$$c_{i}=\frac{1}{T}\int_{0}^{T}{\left|u_{i}\left(t\right)\right|}^{2}dt.$$

Here we have not included intrinsic oscillation (ω) as part of the effort, as all individuals oscillate equally—we only consider the effort to adjust relative to others.

The net payoff $p_{i}$ is defined as the difference between the benefit and the cost, weighted by a relative cost parameter β:

(5) $$p_{i}=b_{i}-\beta c_{i}.$$

## 3 Results

### 3.1 Group Membership Dynamics With Binary Phase Lag

We begin with the simple scenario consisting of two groups of individuals, cooperators and cheaters, each of which comprises a corresponding fraction of the population $n_{\mathrm{co}}$ and $n_{\mathrm{ch}}$ (with $n_{\mathrm{co}}+n_{\mathrm{ch}}=1$). Then the evolutionary dynamics corresponding to the above payoff function Equation (5) satisfies:

(6) $$\begin{array}{cc} n_{\mathrm{co}}^{\left(g+1\right)} & =n_{\mathrm{co}}^{\left(g\right)}\left[1+k_{1}\left(p_{\mathrm{co}}-\bar{p}\right)\right],\\ n_{\mathrm{ch}}^{\left(g+1\right)} & =n_{\mathrm{ch}}^{\left(g\right)}\left[1+k_{1}\left(p_{\mathrm{ch}}-\bar{p}\right)\right], \end{array}$$

where the index g indicates the generation number and $k_{1}$ quantifies the strength of the linear dependence between individual payoff and reproduction chances; $\bar{p}$ is the mean payoff of the population. (See section 1 of the Supporting Information for the derivation of Equation 6.)

The population evolution in Equation (6) represents expected values. It can be implemented with stochastic effects by defining the relative reproductive rate $r_{i}$ to follow the Fermi selection rule [30] as a function of payoff,

(7) $$r_{i}\propto \frac{e^{p_{i}}}{\max_{j}e^{p_{j}}},$$

so that each individual in the next generation has a probability of $r_{i}/\sum_{j}r_{j}$ to be the offspring of individual i.

Taking $k_{1}=k\Delta t$, where k now captures the time scale, we set $n^{\left(g\right)}\mapsto n\left(t\right)$, and $n^{\left(g+1\right)}\mapsto n\left(t+\Delta t\right)$ and let Δt→0 to obtain the continuous‐time deterministic version of Equation (6):

(8) $$\begin{array}{cc} \frac{d}{dt}n_{\mathrm{co}} & =kn_{\mathrm{co}}\left(p_{\mathrm{co}}-\bar{p}\right),\\ \frac{d}{dt}n_{\mathrm{ch}} & =kn_{\mathrm{ch}}\left(p_{\mathrm{ch}}-\bar{p}\right), \end{array}$$

where $\bar{p}=n_{\mathrm{co}}p_{\mathrm{co}}+n_{\mathrm{ch}}p_{\mathrm{ch}}$.

We numerically explore Equations (8) with the interpretation of cooperators as individuals with a phase lag of $\alpha_{\mathrm{co}}=0$ and cheaters as individuals with a phase lag $\alpha_{\mathrm{ch}}\neq 0$ (but equal for all of them—they are identical). We emphasize that here $\alpha_{\mathrm{co}}$ and $\alpha_{\mathrm{ch}}$ are fixed: Selection is applied only to the abundances of group memberships. Another way to view this is that the α values *can* change, but these changes are restricted to exactly two specific values (namely, zero and $\alpha_{\mathrm{ch}}$).

**FIGURE 2:**
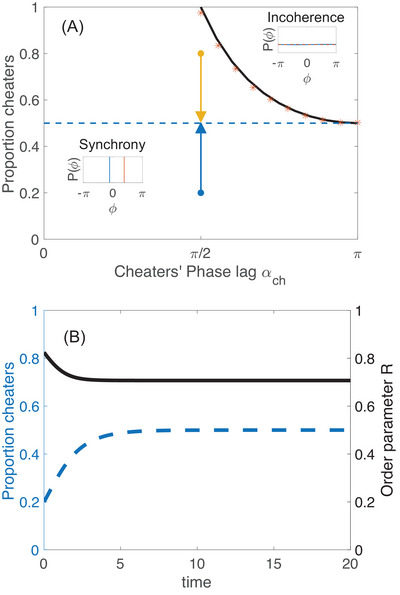
Phase transition for two‐group model. Panel A illustrates a simplified case where the population consists of two distinct types of individuals: cooperators ($\alpha_{\mathrm{co}}=0$) and cheaters, for fixed cheater phase lags $\alpha_{\mathrm{ch}}$ from 0 to π. Under uniform coupling strength and natural frequency, the system exhibits an incoherent state when $\alpha_{\mathrm{ch}}\geq \pi /2$. The theoretical transition boundary (solid black curve) is given by the expression ${\left(1-\cos\left(\alpha_{\mathrm{ch}}\right)\right)}^{-1}$ while red dots represent results from numerical simulations. The blue/yellow dots in Panel A represent the initial population sizes of cheaters, while the dashed blue line shows the equilibrium population of cheaters as a function of $\alpha_{\mathrm{ch}}$. Insets in Panel A show states commonly observed in the long‐term behavior at synchrony and incoherence. Panel B demonstrates the population dynamics driven by competition between cooperators and cheaters—specifically the dynamics for blue arrow in Panel A. Here cheaters (blue dashed line) adopt a phase lag of $\alpha_{\mathrm{ch}}=\pi /2$. The initial condition is set to $n_{\mathrm{ch}}=0.2$, representing the initial cheater fraction.

Figure 2A presents the results of numerical simulations with N=1000 oscillators and illustrates the critical threshold beyond which a population of cheaters can no longer sustain synchrony. Figure 2B shows the dynamical relaxation to equilibrium in a typical case. For persistent populations, the proportion of cooperators and cheaters at equilibrium is always 50−50 (as shown in Figure 2A). This is due to the fact that the minority population loses its advantage as it grows to become half the population.

Simple analytical results can be obtained in this case. Assuming that at equilibrium each subpopulation is synchronized and all oscillators are frequency locked, cooperators share the same phase $\theta_{\mathrm{co}}$ and cheaters share the same phase $\theta_{\mathrm{ch}}$, with $\Delta \theta_{0}=\theta_{\mathrm{ch}}-\theta_{\mathrm{co}}$ constant over time. $n_{\mathrm{co}}$ and $n_{\mathrm{ch}}$ are the fractions of cooperators and cheaters in the population respectively. Substituting these into Equation (1), we obtain:

(9) $$\begin{array}{cc} \frac{d}{dt}\theta_{\mathrm{co}} & =\omega +Kn_{\mathrm{ch}}\sin\left(\Delta \theta_{0}\right),\\ \frac{d}{dt}\theta_{\mathrm{ch}} & =\omega +K\left[n_{\mathrm{co}}\sin\left(-\Delta \theta_{0}+\alpha_{\mathrm{ch}}\right])+n_{\mathrm{ch}}\sin\left(\alpha_{\mathrm{ch}}\right)\right]\;, \end{array}$$

or, expressed fully in the angle difference variable $\Delta \theta_{0}$,

(10) $$K^{-1}\frac{d}{dt}\Delta \theta_{0}=n_{\mathrm{co}}\sin\left(-\Delta \theta_{0}+\alpha_{\mathrm{ch}}\right)+n_{\mathrm{ch}}\sin\left(\alpha_{\mathrm{ch}}\right)-n_{\mathrm{ch}}\sin\left(\Delta \theta_{0}\right).$$

Setting the time derivative to zero, we find the equilibrium solution

$$\Delta \theta_{0}^{*}=\alpha_{\mathrm{ch}}\;.$$

Linear stability analysis reveals that this solution is stable when

(11) $$n_{\mathrm{ch}}<\frac{1}{1-\cos(\alpha_{\mathrm{ch}})},$$

which matches the numerical results shown in Figure 2A. In section 2 of the Supporting Information, we generalize these results to any binary choice of α.

The key message we take away from this model is as follows: It is possible for both cheaters and cooperators to coexist in the population, but there is an upper limit on the number of cheaters the population can sustain. That upper limit is $1/(1-\cos\left(\alpha_{ch}\right))$.

### 3.2 Evolutionary Dynamics With Phase Lag Distributions

In this section, we introduce a new aspect: *mutation*. The inclusion of mutations is to make the evolutionary dynamics more realistic now that we relax the binarization of the phase lags—now α may change across generations. Due to this modification, we need to include the notion of policing. Without policing, everyone in the group will eventually cheat to increase their reproductive success. As a result, the group will not be able to synchronize, which in turn will lead the population to die out.

With policing, we allow the strategy function f(Δθ) to incorporate information beyond mere attractiveness: It also now indicates the optimal balance between maximizing mate attraction and minimizing police intervention (due to, e.g., female choice constraints or actions by competitive males). We note that the payoff primarily depends on the choice of the strategy function f(Δθ), which itself depends only on deviation from the mean phase (since payoff $p_{i}=b_{i}-\beta c_{i}=f_{i}\left(\Delta \theta \right)g\left(R\right)-\beta c_{i}$ where g(R) is uniform over the population and $c_{i}$ is nearly uniform over the population). Other components of the payoff are approximately uniform across individuals at equilibrium due to frequency locking.

We explore the dynamics of the evolution of the population's α distribution numerically. For each generation, to compute individual payoffs at equilibrium, we numerically integrate the dynamics given by Equation (1). After transients have decayed, we use the final phases to compute the payoff of each individual. The reproduction probability for each individual is determined by Equation (7) given the payoff.

To include mutation, a random subset of the new population undergoes mutation on the phase lag α such that:

(12) $$\alpha_{i}^{\left(\text{offspring}\right)}=\alpha_{i}^{\left(\text{parent}\right)}+I_{i}\varepsilon_{i},$$

where

$$I_{i}=\left\{\begin{array}{cc} 1 & \text{if}\;i\in \text{mutation set}\\ 0 & \text{otherwise}, \end{array}\right.$$

and $\varepsilon_{i}∼N\left(0,\sigma_{\text{mutate}}\right)$ represents the mutation perturbation.

**FIGURE 3:**
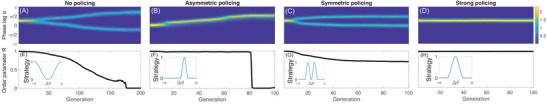
Evolutionary dynamics with continuous phase lag distribution. Heatmaps in upper panels show probability density for each phase lag α over generations; lower panels illustrate evolution of the corresponding order parameter R. Panels A and E: no effective policing and initial condition (IC) is N(π/4,0.1). Panels B and F: asymmetric policing, IC is N(0,0.1). Panels C and G: symmetric policing function, IC is N(π/4,0.1). Panels D and H: strong policing function, IC is N(π/4,0.1). Insets in lower panels show the overall strategy function resulting from a fixed symmetric attraction function and the given policing (see Figure 1B). All trials had N = 1000 oscillators, $\sigma_{\text{mutate}}=0.05$, and $n_{\text{mutate}}=0.1$.

In Figure 3, we initialize the population's phase lag distribution as a normal distribution with mean μ and standard deviation σ. We consider four cases, all under the assumption that the attracted individuals have no preference for phases advanced versus delayed relative to the group (symmetric attraction function). The four scenarios differ in how policing is applied through the tolerance function: (1) no policing (without any tolerance function), (2) asymmetric policing (e.g., only individuals lagging behind the group are penalized), (3) symmetric policing (individuals both ahead of and behind the group are penalized), and (4) strong policing (individuals even slightly away from the mean group phase are penalized to such an extent that there is no net benefit to being out of sync, despite mate attraction being minimized when in sync).

Figure 3A shows results for the “no policing” scenario. There individuals are incentivized to cheat as much as possible to maximize their individual benefit. This leads to a breakdown of coordination, and the population ultimately goes extinct due to the resulting low level of synchrony. Figure 3B shows results for asymmetric policing, and more specifically, for the case where only individuals lagging behind the group are penalized. Here the distribution of phase lags α drifts in the positive direction, with the mean eventually approaching π/2, when a breakdown of synchrony occurs and the population goes extinct. In Figure 3C, symmetric policing is imposed on both individuals ahead and behind the group. We observe that the population splits into two clusters and stabilizes.

Interestingly, this symmetry in the policing function appears to be necessary for survival of the population. Asymmetry leads eventually to takeover by cheaters, and thus populations that survive, according to this model, must find a way to impose a symmetric cost on either leading or lagging cheaters.

Finally, Figure 3D illustrates the case of strong policing, where the incentive to cheat is entirely eliminated: The cost of deviating from the group outweighs any potential benefit. In this regime, unsurprisingly, the population remains stable and synchronous; the system is very similar to the case with no cheaters.

We note that the long‐term survival of the population depends on the interplay between the choice of the policing function and the initial conditions μ,σ. If the policing is too weak (i.e., the gap between the peaks in the strategy function is too wide) and/or the initial population is not collaborative enough (i.e., μ is too far from 0), the population will fail to survive—see section 2.1 of the Supporting Information for exact limits.

Additional comments on the robustness of our results can be found in sections 3 and 5 of the Supporting Information.

### 3.3 Simulation Details

#### 3.3.1 Binary Phase Lag Simulation

We start with the simple case where we only allow the phase lag to take on two given values: $\alpha_{\mathrm{co}}$ and $\alpha_{\mathrm{ch}}$, for cooperators and cheaters, respectively. The population sizes of cooperators and cheaters evolve according to Equation (8). The simulation results are deterministic given the initial conditions.

We use the following functions to calculate the payoff:

$$\begin{array}{cc} & g(R)=R,\\ & f(\Delta \theta )=1-\cos(\Delta \theta ), \end{array}$$

where Δθ=θ−ϕ. These are simple choices consistent with our assumptions that g(R) be a monotonically increasing function of R and f(Δθ) be a 2π periodic function that penalizes individuals with small phase deviations.

There are two possible equilibrium states of the population. When $n_{\mathrm{ch}}<{\left(1-\cos\alpha_{\mathrm{ch}}\right)}^{-1}$, all cooperators have the same phase $\theta_{co}$ and all cheaters have same phase $\theta_{ch}$ with $\theta_{ch}-\theta_{co}=\alpha_{\mathrm{ch}}$ at equilibrium. In this case, $p_{\mathrm{co}}$, $p_{\mathrm{ch}}$, and $\bar{p}$ can be calculated explicitly as

$$\begin{array}{cc} p_{\mathrm{co}} & =R(1-\cos(\phi )),\\ p_{\mathrm{ch}} & =R(1-\cos\left(\alpha_{\mathrm{ch}}-\phi \right)),\\ \bar{p} & =n_{\mathrm{co}}p_{\mathrm{co}}+n_{\mathrm{ch}}p_{\mathrm{ch}}, \end{array}$$

with $\exp\left(i\phi \right)=n_{\mathrm{ch}}\exp\left(i\alpha_{\mathrm{ch}}\right)+n_{\mathrm{co}}$ (ϕ defined as in Equation 2).

Note that we are free to remove the cost term from the payoff here, since the cost is the same for all individuals in this scenario, and thus removing it does not change the value of $\left(p_{\mathrm{co}}-\bar{p}\right)$ or $\left(p_{\mathrm{ch}}-\bar{p}\right)$.

We do not simulate the case when $n_{\mathrm{ch}}>{\left(1-\cos\alpha_{\mathrm{ch}}\right)}^{-1}$ since the order parameter R approaches zero in this regime, indicating that the population cannot maintain synchrony and thus lacks sufficient fitness for reproduction, ultimately leading to extinction.

To numerically determine the critical boundary, we integrate Equation (1) using various initial population compositions and values of $\alpha_{\mathrm{ch}}$. We sweep $\alpha_{\mathrm{ch}}$ from 0 to π. For each value of $\alpha_{\mathrm{ch}}$, we conduct a series of simulations with increasing initial fractions of cheaters $n_{\mathrm{ch}}\left(t=0\right)$. The critical proportion of cheaters $n_{\mathrm{ch}}^{(}\mathrm{crit})$ is identified as the smallest cheater fraction that causes the system to become incoherent at equilibrium. This procedure yields a series of $\left(\alpha_{\mathrm{ch}},n_{\mathrm{ch}}^{(}\mathrm{crit})\right)$ pairs and, as shown in Figure 2, they appear to follow the curve $n_{\mathrm{ch}}^{(}\mathrm{crit})={\left(1-\cos\alpha_{\mathrm{ch}}\right)}^{-1}$, consistent with the analytical prediction.

#### 3.3.2 Population Dynamics Simulation With Mutation

In these simulations we allow the individual phase lags to mutate across generations. A key practical distinction is that we can obtain a closed‐form expressions for equilibrium payoff in the binary case, but we cannot do so in the model with mutation: Payoff needs to be evaluated numerically. For each generation, we numerically integrate the dynamics given by Equation (1) up to $T_{\mathrm{final}}=100$ with N=1000 individuals. Without loss of generality, we choose ω=0 and K=1.

For payoffs with symmetric policing, we choose

$$\begin{array}{cc} & g(R)=R,\\ & f\left(\Delta \theta \right)=\left\{\begin{array}{cc} \cos\left[4\left(\Delta \theta -\frac{\pi }{4}\right)\right]+1, & \left|\Delta \theta \right|<\frac{\pi }{2}\\ 0, & \left|\Delta \theta \right|\geq \frac{\pi }{2} \end{array}\right.. \end{array}$$

For payoffs with asymmetric (one‐side) policing, we choose

$$\begin{array}{cc} & g(R)=R,\\ & f\left(\Delta \theta \right)=\left\{\begin{array}{cc} \cos\left[4\left(\Delta \theta -\frac{\pi }{4}\right)\right]+1, & 0<\Delta \theta <\frac{\pi }{2}\\ 0, & \text{otherwise} \end{array}\right.. \end{array}$$

In both cases we set β=1 to calculate the payoff. The choice of β is not critical as cost $c_{i}$ is nearly identical across individuals. The payoff for each individual is calculated with Equation (5) averaged over the final 10% of the simulation time.

In our simulations, we randomly select 10% ($n_{\text{mutate}}=0.1$) of the population to mutate at each generation and model those mutations via additive perturbations of magnitude $\varepsilon_{i}∼N\left(0,\sigma_{\text{mutate}}\right)$ as shown in Equation (12). We use $\sigma_{\text{mutate}}=0.05$, since smaller $\sigma_{\text{mutate}}$ helps keep the resultant dynamics smooth. The magnitude of these perturbations controls the rate at which the distribution of α drifts across generations.

## 4 Discussion and Conclusions

We have developed a novel approach based on the Sakaguchi–Kuramoto model where we swap the usual heterogeneity of natural frequencies for a different source of irregularity: a distribution of phase lags α (usually taken to be a constant, although see some exceptions: [31, 32, 33, 34, 35, 36]). Phase lags can take on different interpretations, including individual delays (e.g., due to computation times internal to each agent) or, as we have considered here, purposeful attempts at misalignment with others. Since multiple species are known to synchronize while lekking (e.g., fireflies [37], crickets [38], frogs [39, 40, 41], and crabs [5]—see [1] for a review on the topic), we think our model has the potential for wide application.

Our main result is the discovery that populations of both cooperators and cheaters can coexist even in a highly simplified model, and we analytically derive an upper bound on the fraction of cheaters. This suggests an explanation for why natural animal collectives may indeed tolerate a subset of cheaters. Importantly, these conclusions are robust to reasonable choices of strategy functions and other parameters: The evolutionary equilibrium is controlled primarily by the shape of the policing function. In particular, symmetric policing—where straying ahead or behind the pack is enforced equally—is the only stable option in the presence of cheaters. Thus, if policing is observed in a natural system, we predict it will be applied equally to both early and late signalers (though this poses the natural follow‐up question of what stabilization mechanisms could exist to enforce such symmetry).

Another point to bear in mind regarding this system is that it is difficult to distinguish cheaters from cooperators based solely on the phase distribution, as cheaters tend to form one synchronized cluster while cooperators form another in the steady state. This may be because both groups incur the same coupling cost in our model, given the universal coupling strength K. However, if cheaters and cooperators experience different coupling costs, it may become possible to differentiate them from the phase distribution, as suggested in [42].

Although the model we analyze here has multiple parameters, the number of effective control parameters is actually very limited. The only parameters that strongly influence the outcomes are those related to the strategy function. See section 4 of the Supporting Information for a detailed discussion of the relative influence of all the parameters.

A limitation of our study is that we use a global order parameter R to quantify synchrony, which does not account for clusters of phases. For example, when the population splits into two phase clusters, a perfect cooperator may not respond to the global mean phase, but rather to the mean phase of their local cluster.

It is also worth mentioning that we focus here on synchrony that arises among intrinsically oscillatory individuals, as opposed to synchrony related to, for example, feeding [43] or circadian rhythms [44]. Though such examples do show simultaneous activity, they likely have a different origin connected to environmental cues (e.g., entrainment to the light‐dark cycle) or work coordination [45]. Similarly, we would expect different evolutionary origins for cases of synchrony in eusocial organisms [44, 45].

We can, though, situate this work within the broader context of frequency‐locking behavior beyond just in‐phase relationships. Some species may be hard‐wired to cheat, in the sense that they attempt to avoid overlap in calls when in small groups (e.g., some frog species [41]). This raises other interesting modeling questions due to the potential for frustration effects where no individual can fully achieve its signal timing goals.

Future work could focus on generalizations of our model. For simplicity we kept the model in an all‐to‐all setup. Of course the positions of the males in space (whether in 1‐, 2‐ or 3D) could be important. It is easy to imagine that the relative positioning of cheaters, be it within clusters or more uniformly distributed across the group, could influence the overall dynamics. For instance, selective attention and local avoidance strategies have been observed in frog choruses, which can also disrupt full synchronization [46, 47]. Beyond spatial considerations, it would also be valuable to shed light on what actually determines the time scales associated with these evolutionary dynamics.

We conclude with a hope that our model could offer testable predictions for real animal populations. To that end, it is crucial that quality detailed measurements are conducted, where continuous individual activity (call‐by‐call) within collectives is documented for long durations. Ideally, when analyzing pairwise phase differences, some nearby neighbors will appear to have consistent nonzero phase differences. If indeed cheaters are well defined and detected, then a rigorous quantification of their fraction within the group could be assessed, yielding insight into the real‐world utility of our model.

## Author Contributions

G.A. conceived the idea of the study; G.A. and D.M.A. obtained funding; G.A., R.G, and D.M.A. devised the model; R.G. implemented and analyzed the model with input and supervision from G.A. and D.M.A.; all authors wrote the paper and revised it.

## Conflicts of Interest

The authors declare no potential conflicts of interest.

## Supporting information



Supporting Information.pdf

## Footnotes

1 Of course, this is not the only option; also for instance antiphase relationships may be seen as a form of sync.

2 This is the phase of oscillator i relative to the mean group phase, and we will later denote it by Δθ.
