# Supplementary material for: Modeling the Evolution of Collective Synchrony
Source: Ann N Y Acad Sci. 2026 Mar 3;1557(1):e70226. doi: 10.1111/nyas.70226 (PMC12955760; doi:10.1111/nyas.70226)
Supplement: Supplementary file 1 — Supporting Information.pdf [file NYAS-1557-0-s001.pdf]

Supporting information: Modeling the evolution of collective synchrony

Guy Amichay*, Ruoming Gong*, Daniel M. Abrams

1 Derivation of two-group equations

We assume that the expected number of offspring for an individual with payoff p_i, in a population with mean payoff $\bar{p}$, is linearly proportional to that difference $p_i - \bar{p}$. So after g generations, if there are $N_i^{(g)}$ individuals descended from an initial progenitor i, the number of new offspring (in expected value) will be

$$N_i^{(g+1)} = (1-\gamma)N_i^{(g)} + k_0 N_i^{(g)}(p_i - \bar{p}), \tag{1}$$

where k_0 is a constant of proportionality for birth and γ determines the fraction of individuals who die in a single generation timescale.

Rewriting in terms of the fractional population $n_i^{(g)} = N_i^{(g)}/N^{(g)}$ where $N^{(g)} = \sum_i N_i^{(g)}$, we get

$$n_i^{(g+1)} N^{(g+1)} = n_i^{(g)} N^{(g)} \left[1 - \gamma + k_0(p_i - \bar{p})\right]. \tag{2}$$

To enforce that $\sum_i n_i^{(g)} = 1$ in all generations (so n represents a fraction as intended), the ratio $N^{(g+1)}/N^{(g)} = 1-\gamma$ is required. Then this simplifies to

$$n_i^{(g+1)} = n_i^{(g)} \left[1 + \frac{k_0}{1-\gamma}(p_i - \bar{p})\right]. \tag{3}$$

We can define $k_1 = \frac{k_0}{1-\gamma}$ as an effective generational growth rate for each subpopulation i.

In the simple case where there are only two types of individuals present in the population, this simplifies to two coupled equations. Considering the two types as cooperators and cheaters, the system becomes

$$\begin{aligned} n_{\text{co}}^{(g+1)} &= n_{\text{co}}^{(g)} \left[1 + k_1(p_{\text{co}} - \bar{p})\right], \\ n_{\text{ch}}^{(g+1)} &= n_{\text{ch}}^{(g)} \left[1 + k_1(p_{\text{ch}} - \bar{p})\right], \end{aligned} \tag{4}$$

where $n_{\text{co}}, n_{\text{ch}}$ represents the fraction of the population of cooperators and cheaters respectively, and the index g indicates the generation number.

2 Linear stability analysis of the binary phase lag model

Assume at equilibrium that the whole population is frequency locked. The cooperators all have phase θ_{co}, and the cheaters all have phase θ_{ch}, with $\Delta\theta_0 = \theta_{\mathrm{ch}} - \theta_{\mathrm{co}}$ constant over time. n_{co} and n_{ch} are the percentages of cooperators and cheaters in the population respectively ($n_{\mathrm{co}} + n_{\mathrm{ch}} = 1$). Without loss of generality, we can set $\omega = 0$ and $K = 1$. Then we have (in a co-rotating frame)

$$\dot{\theta}_{co} = n_{\mathrm{ch}} \sin(\theta_{\mathrm{ch}} - \theta_{\mathrm{co}} + \alpha_{\mathrm{co}}) - n_{\mathrm{ch}} \sin(\alpha_{\mathrm{ch}}),$$
$$\dot{\theta}_{ch} = n_{\mathrm{co}} \sin(\theta_{\mathrm{co}} - \theta_{\mathrm{ch}} + \alpha_{\mathrm{ch}}) - n_{\mathrm{co}} \sin(\alpha_{\mathrm{co}}).$$

The two eigenvalues of the Jacobian matrix for this system are:

$$\lambda_1 = 0, \quad \lambda_2 = -n_{\mathrm{co}} \cos(\alpha_{\mathrm{co}}) - n_{\mathrm{ch}} \cos(\alpha_{\mathrm{ch}}).$$

Here λ_1 is the eigenvalue corresponding to the direction $\theta_{ch} - \theta_{co} = \alpha_{\mathrm{ch}} - \alpha_{\mathrm{co}}$, i.e., perturbations that preserve the phase difference between cheaters and cooperators; the system is neutral to any perturbation in this direction, which simply reflects the freedom of choice of phase coordinate origin.

Setting $\lambda_2 < 0$, we find

$$n_{\mathrm{ch}} < \frac{\cos(\alpha_{\mathrm{co}})}{\cos(\alpha_{\mathrm{co}}) - \cos(\alpha_{\mathrm{ch}})}. \tag{5}$$

In particular, when $\alpha_{\mathrm{co}} = 0$ and $\alpha_{\mathrm{ch}} = \alpha$, this reduces to the stability condition

$$n_{\mathrm{ch}} < \frac{1}{1 - \cos(\alpha)}.$$

This sets an upper bound on the proportion of cheaters that may be present in a synchronous population.

2.1 Connection between policing and initial cooperativeness

Here we comment on the connection between the strategy function and the initial population distribution for long-term stability of the population.

Suppose σ_{mutate} is small and the gap between the two modes of the strategy function is ℓ. We observe that the population eventually stabilizes into two distinct subpopulations. The separation between the two modes of the strategy function approximately matches the phase lag difference between the two stabilized subpopulations, as individuals maximizing their benefits. Because the mutation process has zero mean (no directional bias for α), the two subpopulations are expected to be equal in size and symmetrically located about the initial α distribution's mean μ (without loss of generality we choose $\mu > 0$). That is, $\mu_1 = \mu - \ell/2$ and $\mu_2 = \mu + \ell/2$. We focus on the case where $\mu_1 \in (-\pi/2, \pi/2), \mu_2 \in [\pi/2, 3\pi/2]$, which corresponds to parameter regimes in which the population can persist in the long term.

Assuming that the standard deviations of the two subpopulations are small, the stability condition can be approximated using Eq. 5, with $\alpha_{\mathrm{co}} = \mu_1, \alpha_{\mathrm{ch}} = \mu_2, n_{\mathrm{ch}} = 1/2$. This leads directly to the condition:

$$\frac{\cos(\mu - \frac{\ell}{2})}{\cos(\mu - \frac{\ell}{2}) - \cos(\mu + \frac{\ell}{2})} \gtrsim \frac{1}{2}. \tag{6}$$

3 Robustness

3.1 Robustness: discrete vs. continous evolution

For the model with binary α, we check that results from simulations with discrete generations (main text Eq. (6)) are consistent with those from equivalent simulations with continuous evolution (main text Eq. (8)). In Figure 1, as expected, the discrete simulation appears as a noisier version of the continuous one, with the overall picture remaining consistent.

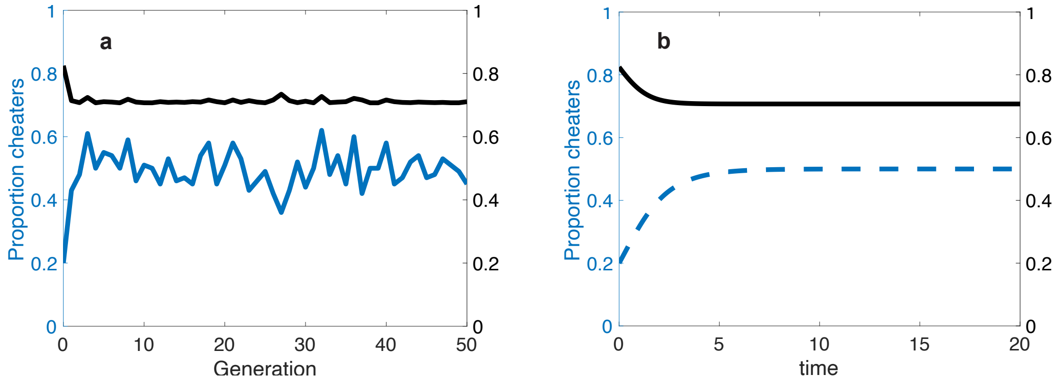

Figure 1: **Comparing discrete and continuous evolution equations.** Cooperators are characterized by a phase lag of $\alpha_{\rm co} = 0$, while cheaters adopt a phase lag of $\alpha_{\rm ch} = \pi/2$. When cooperators are the majority, cheaters gain a reproductive advantage by deviating from the mean phase of the population. Conversely, when cheaters become dominant, cooperators—now the minority—regain a fitness advantage. This interplay creates cyclic fluctuations in population composition, oscillating around a mean of 50%. When population changes occur smoothly, the dynamics stabilize at an even split between cooperators and cheaters.

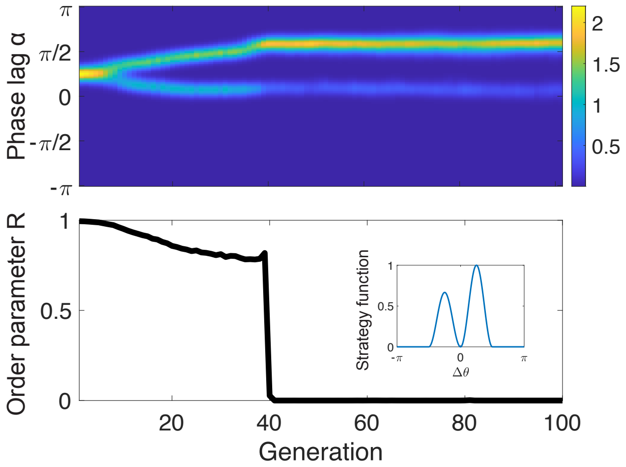

Figure 2: **Asymmetric policing.** When policing is asymmetric, it yields an asymmetric strategy function that rewards individuals ahead of the pack differently from those behind. In such cases, the population cannot sustain itself over the long term and eventually goes extinct.

3.2 Robustness: asymmetric bimodal strategy function

We examine numerically whether a symmetric choice of the policing function is essential for the long-term survival of the population. Specifically, in Fig. 2, we consider an asymmetric bimodal strategy function and

observe that the subpopulation with negative phase lag eventually dies out due to the accumulated advantage of those with positive phase lag. As a result, the entire population fails to achieve synchronization.

3.3 Robustness: nonzero payoff for perfect cooperators

We examine the robustness of the simulation results when perfect cooperators ($\alpha = 0$) receive a nonzero payoff. As shown in Fig. 3, the results exhibit the same qualitative behavior as those in Fig. 3(c) of the main text, where perfect cooperators receive zero payoff.

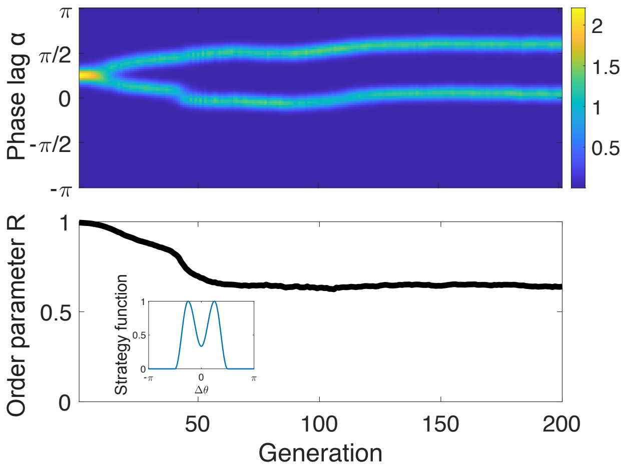

Figure 3: **Nonzero payoff at $\alpha = 0$.** To test the robustness of our choice to have strategy function drop to zero at $\Delta\theta = 0$, we simulate an alternative strategy function with a non-zero value at $\Delta\theta = 0$, as shown in the inset. The resulting population dynamics remain qualitatively unchanged as compared to main text Fig. 3(c), suggesting that the model is not sensitive to the specific value of the strategy function at $\Delta\theta = 0$, as long as the overall symmetry is preserved.

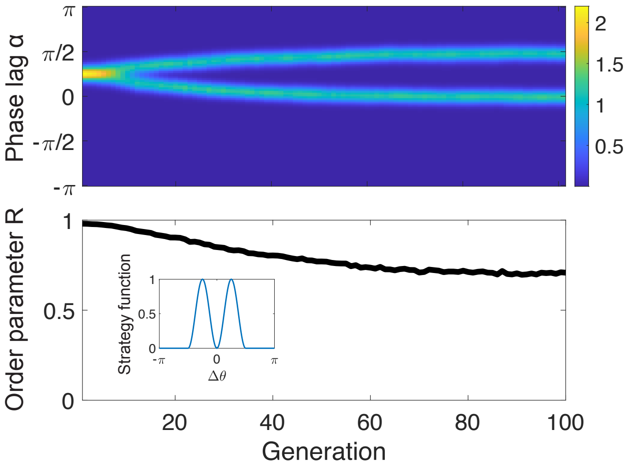

Figure 4: **Natural frequency heterogeneity.** To test the robustness of the simulation results to heterogeneity in the natural frequency distribution, instead of identical natural frequencies, we choose $\omega \sim \mathcal{N}(\mu_\omega, \sigma_\omega)$ with $\mu_\omega = 0$ and $\sigma_\omega = 1$. The resulting population dynamics appears qualitatively unchanged when compared with main text Fig. 3(c), as long as the coupling strength is above a critical value.

3.4 Robustness: natural frequency heterogeneity

We examine the robustness of the simulation results when the population has a heterogeneous natural frequency distribution $\omega \sim \mathcal{N}(\mu_\omega, \sigma_\omega)$ with nonzero σ_ω. As shown in Fig. 4, the results appear to exhibit the same qualitative behavior as those in Fig. 3(c) of the main text, where there is zero heterogeneity in natural frequency.

4 Table of parameters used in simulation

While our model may appear to contain a large number of parameters, we believe that it is actually remarkably simple at heart. There are very few "effective control parameters" that have an impact on the model's qualitative behavior; the only parameters that strongly influence the outcomes are those related to the strategy function f. As the insets in main text Fig. 3 show, we examine several options for this function, and find that the symmetry properties do matter, but we purposefully do not introduce any tunable parameters into our formulas (shown in main text section 3.3.2). Clearly the amplitude of f cannot have an influence since it would be redundant with β and k, and we explore what we believe to be all the qualitatively different (yet plausible) cases for number and symmetry of peaks. Of course the details of the algebraic formulas might make a small difference in the details of the system evolution but cannot change the "big picture" behavior.

**Parameter**	**Description**	**Value used in simulations**
N	Number of oscillators	1000
K	Coupling strength	1
β	Relative cost parameter (balances cost vs. benefit—see main text Eq. (5))	1
ω	Natural frequency	0
T_{final}	Fast time scale simulation time	100
k	Evolution time scale	1
σ_{mutate}	Standard deviation of mutation distribution	0.05
n_{mutate}	Proportion of population undergoing mutation at each generation	0.1
$\alpha(t=0)$	Initial phase lag	various choices
$g(R)$	Attraction function	$g(R) = R$
$f(\Delta\theta)$	Tolerance function	various choices

Table 1: **Parameters used in simulations.**

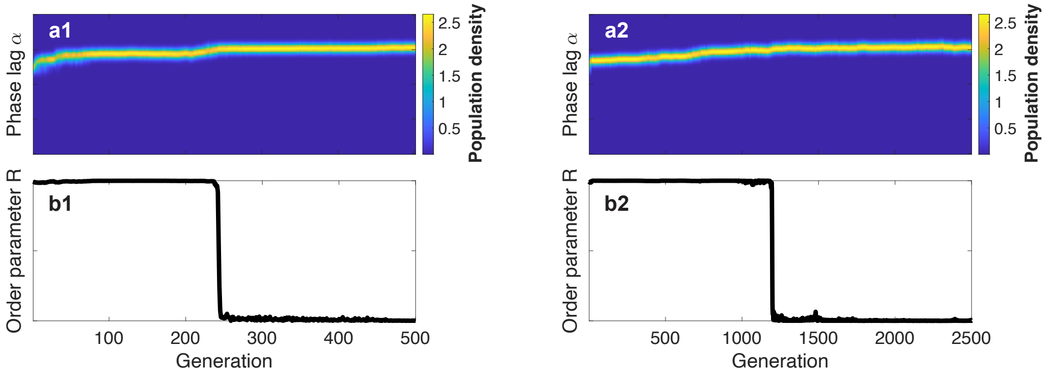

Figure 5: **Examples of simulations with different mutation rates.** Simulations with asymmetric policing function as in main text Fig. 3(b) and (f), but with different σ_{mutate}. In the upper panels (a1) and (b1), we chose $\sigma_{\text{mutate}} = 0.05$. In the lower panel (a2) and (b2), we chose $\sigma_{\text{mutate}} = 0.025$. Unlike main text Fig. 3, here, in each generation, only one individual undergoes mutation. Mutation rates do not affect the equilibrium state but determine the timescale to reach equilibrium. Both simulations had $N = 100$ oscillators and $n_{\text{mutate}} = 1/100$.

In Table 1 we summarize all parameters. The relative cost coefficient β plays almost no role since costs are nearly uniform over individuals. The expected number of offspring $g(R)$ is assumed to be a monotonically increasing function of order R, and taken to be the parameter-free linear function $g(R) = R$

in our formulation. Coupling strength K and natural frequency ω are both held constant—and in a system of nonidentical oscillators the ratio of K to σ_ω is important, but in most of our simulations we assume identical oscillators, so only the fact that $K > 0$ matters (other than setting a reference time for fast time scale dynamics).

The parameter k sets the time scale for evolution. T_{final} is simply chosen such that equilibrium is reached on the fast time scale. Finally, mutation rate σ_{mutate}, and proportion mutating in each generation n_{mutate}, together set the reference time for slow time scale dynamics. We have conducted additional simulations where $n_{\text{mutate}} = 1/N$ (only one individual mutates at each slow-time simulation step, see Fig. 5) to demonstrate that it could be entirely removed from the implementation of our model (and would in fact lead to smoother dynamics), but we retain it in some simulations because it allows for faster simulation (faster convergence to equilibrium) at the cost of more numerical noise[1].

To summarize, we believe the results are robust and sensitive only to a very small number of modeling choices. In creating simulations, however, we had to introduce additional parameters that could not easily be combined (e.g. any simulation must have at minimum start time, an initial condition, and an end time, but these choices should not be considered as truly relevant to a given model except in that they must be appropriate for the model dynamics to play out), but these do not play a major role in the model dynamics.

[1]Higher mutation rates in general lead to faster convergence to equilibrium, but with more numerical noise.

5 Binary case with noise

To evaluate robustness of results in our binary-group simulations, we introduce "quenched" noise in both the natural frequencies ω and phase lags α. Note that the noise in phase lags is introduced only for cooperators, who are given fixed α values drawn from a normal distribution $\mathcal{N}(0, \sigma_\alpha)$; cheaters still have a single universal phase lag α_{ch}. The system is then simulated until it reaches equilibrium (no slow-time evolution).

As shown in Figs. 6 and 7, increasing heterogeneity in these quantities (either ω or α) decreases the maximum proportion of cheaters that can be sustained in the population (perhaps because of the reduction in synchronous order associated with having non-identical oscillators even in the cooperating group).

We also emphasize that we focus on regimes where cheaters have a phase lag much larger than the typical fluctuations, i.e., $\alpha_{\text{ch}} \gg \sigma_\alpha$; otherwise, it becomes difficult to distinguish deliberate cheating from natural variability.

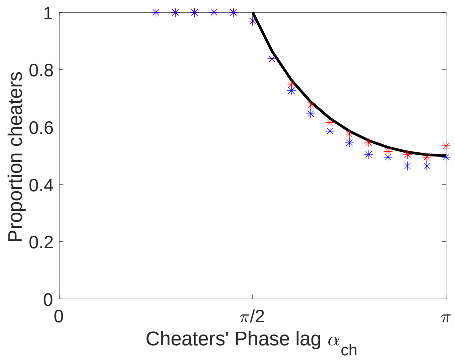

Figure 6: **Phase transition for a two-group model with heterogeneous phase lags.** We consider the simplified case with two behavioral classes similar to main text Fig. 2, but with heterogeneous phase lags for collaborating individuals following $\alpha_i \sim \mathcal{N}(0, \sigma_\alpha)$. We set $\alpha_{\text{ch}} \gg \sigma_\alpha$ so that variation in phase lag among collaborators is distinguishable from deliberate cheating. Red: $\sigma_\alpha = 0.1$, blue: $\sigma_\alpha = 0.5$. The black curve reproduces the theoretical bound from the homogeneous case (identical natural frequencies and only two possible phase lags).

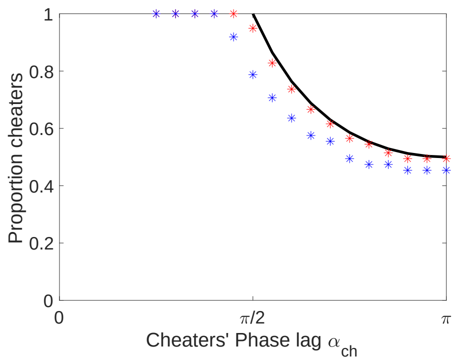

Figure 7: **Phase transition for two-group model with heterogeneous natural frequencies.** We consider the simplified case with two behavioral classes similar to main text Fig. 2, but with heterogeneous natural frequencies for each individuals following $\omega_i \sim \mathcal{N}(0, \sigma_\omega)$. Red: $\sigma_\omega = 0.1$, blue: $\sigma_\omega = 0.5$. The black curve reproduces the theoretical bound from the homogeneous case (identical natural frequencies and only two possible phase lags).
